# Carnosine Protects against Cerebral Ischemic Injury by Inhibiting Matrix-Metalloproteinases

**DOI:** 10.3390/ijms22147495

**Published:** 2021-07-13

**Authors:** Eun-Hye Kim, Eun-Sun Kim, Donggeun Shin, Donghyun Kim, Sungbin Choi, Young-Jun Shin, Kyeong-A Kim, Dabi Noh, Ahmet B. Caglayan, G.K. Rajanikant, Arshad Majid, Ok-Nam Bae

**Affiliations:** 1 College of Pharmacy, Institute of Pharmaceutical Science and Technology, Hanyang University, Ansan 15588, Korea; rladmsgp615@naver.com (E.-H.K.); ikhhycs@hanmail.net (E.-S.K.); stk0987@naver.com (D.S.); ssks7787@naver.com (D.K.); hjklkl123@naver.com (S.C.); tlsdid24@naver.com (Y.-J.S.); hosangkka@naver.com (K.-A.K.); ekqlg@naver.com (D.N.); 2 Department of Physiology, Faculty of Medicine, Istanbul Medipol University, 34810 Istanbul, Turkey; abcaglayan@medipol.edu.tr; 3 School of Biotechnology, National Institute of Technology Calicut, Calicut 673601, India; rajanikant@nitc.ac.in; 4 Sheffield Institute for Translational Neuroscience, University of Sheffield, Sheffield S10 2TN, UK

**Keywords:** ischemic stroke, matrix-metalloproteinases, carnosine, brain edema, tight junction

## Abstract

Stroke is one of the leading causes of death and disability worldwide. However, treatment options for ischemic stroke remain limited. Matrix-metalloproteinases (MMPs) contribute to brain damage during ischemic strokes by disrupting the blood-brain barrier (BBB) and causing brain edemas. Carnosine, an endogenous dipeptide, was found by us and others to be protective against ischemic brain injury. In this study, we investigated whether carnosine influences MMP activity. Brain MMP levels and activity were measured by gelatin zymography after permanent occlusion of the middle cerebral artery (pMCAO) in rats and in vitro enzyme assays. Carnosine significantly reduced infarct volume and edema. Gelatin zymography and in vitro enzyme assays showed that carnosine inhibited brain MMPs. We showed that carnosine inhibited both MMP-2 and MMP-9 activity by chelating zinc. Carnosine also reduced the ischemia-mediated degradation of the tight junction proteins that comprise the BBB. In summary, our findings show that carnosine inhibits MMP activity by chelating zinc, an essential MMP co-factor, resulting in the reduction of edema and brain injury. We believe that our findings shed new light on the neuroprotective mechanism of carnosine against ischemic brain damage.

## 1. Introduction

Ischemic stroke is associated with high morbidity and mortality [1,2,3]. It also contributes to the social burden of disability that persists after strokes. Tissue-type plasminogen activator (tPA) and intra-arterial thrombectomy have been used to treat ischemic stroke [4,5,6]. However, these approaches have limitations due to a narrow therapeutic time window of several hours and side effects such as bleeding [7,8,9,10]. There is an urgent need for the development of a novel therapeutic agent for ischemic stroke. Cerebral edema can be a complication of cerebral infarction. The incidence of cerebral edema has been reported to range from 10–54% in stroke patients and can contribute significantly to irreversible injury [11,12]. Not all stroke patients develop significant edema. In those that do, usually large strokes, edema contributes significantly to morbidity and mortality [13,14,15]. Reducing edema has been proposed as an important therapeutic maneuver that may reduce morbidity and mortality and in severe cases, it may also reduce the utilization of surgical treatments, such ventricular drains and hemicraniectomy. Tissue change caused by edema-induced compression results in neurological deterioration [15]. Cell swelling may not occur in mild (non-disabling) strokes, and the reduction in cerebral blood flow required to induce cell swelling is thought to be more severe than that required to produce acute neurological symptoms [16,17,18]. An imbalance of ions, such as sodium, leads to intracellular osmosis and can cause water to accumulate and cause the cells to swell [19]. Vascular permeability increases due to the destruction of cells constituting the blood-brain barrier, and a consequent increase in osmotic pressure in the extracellular space induces brain edema [20,21]. Furthermore, brain edema caused by capillary endothelial dysfunction leads to the breakdown of the blood-brain barrier (BBB) [22,23]. Tight junction (TJ) proteins, a major component of the BBB, play an important role in sealing the extracellular space. TJ proteins are mainly composed of transmembrane proteins such as claudins, and multiprotein junctional complexes such as zonula occludens (ZOs). TJ proteins contribute significantly to the overall integrity of the BBB [24,25]. TJ proteins of the BBB can be disrupted during ischemic stroke, allowing more vascular-derived substances into the brain [26,27,28]. TJ proteins were also found to be decreased and degraded in brain endothelial cells following ischemic insult of oxygen-glucose deprivation (OGD) [29,30].

Activities of matrix metalloproteinase-2 and -9 (MMP-2 and MMP-9) significantly increased during ischemic stroke in both animal models and patients [31,32,33]. MMP-2 and MMP-9 are calcium-dependent zinc-containing endopeptidases that belong to the MMP family. MMPs are thought to play an important role in cell behavior, such as proliferation, migration (adhesion or dispersion), differentiation, angiogenesis, apoptosis, and host defense [33,34,35,36,37]. MMPs are involved in the disruption of the extracellular matrix in ischemic strokes, which leads to changes in the blood vessel wall and may loosen the matrix around cells, facilitating cellular swelling [38]. High MMP levels in the blood during the acute phase of an ischemic stroke raises the risk of secondary bleeding, which contributes to hemorrhagic transformation [39]. Furthermore, increased MMP expression has been linked to disruption of BBB permeability and the formation of brain edema following focal cerebral ischemia [40]. A previous study found that pharmacological inhibition of MMPs reduces brain edema after focal cerebral ischemia in rats [41]. Increased MMP activity in strokes results in cleavage and disruption of TJ proteins, contributing to brain damage. The loss of TJ protein was reduced when MMP activity was inhibited after focal ischemia [42,43,44].

Since ischemic stroke injury has been associated with multiple simultaneous and sequential pathological processes, there have been considerable efforts to develop drug candidates that have multiple mechanistic targets. For example, biphalin, a non-selective opioid receptor agonist, has exhibited protective effects against ischemic stroke by reducing cerebral infarct volume, cerebral edema, and neurological deficits in animal models. It also reduces protein kinase C-mediated neuronal cell volume increase [45,46,47,48,49]. We and others have previously shown that carnosine, an endogenous dipeptide composed of alanine and histidine, has a protective effect against ischemic brain injury using animal and cellular models of stroke. Improvement in behavioral and histological outcomes in rat/mouse models of ischemic stroke, direct protection against ischemic injury in primary neurons and astrocytes, and modulation of ischemic cell death mechanisms such as excessive autophagy supports a neuroprotective function of carnosine in ischemic stroke [29,31,50,51,52]. Carnosine is known to have pleiotropic biological activities, such as antioxidant, anti-inflammatory, improving muscle function, and reducing neurological impairment. Carnosine administered intravenously alleviated brain injury in both permanent and transient ischemic rat models [50]. Furthermore, carnosine has been shown to have a clinically broad treatment period, with no significant side effects or toxicity [29]. We previously found that pre-treated carnosine inhibits MMPs in a mouse model of focal ischemia [31]. However, no studies have been conducted to determine the mechanism by which carnosine inhibits MMPs or whether it has a direct effect on edema.

The purpose of this study was to investigate if carnosine treatment could reduce infarct volume and brain edema caused by ischemic stroke. The effect of carnosine on MMP activity, which contributes to edema formation, was investigated using brain homogenate zymography and in vitro enzyme assays. The inhibition of tight junction protein degradation in brain endothelial cells by carnosine following ischemic stimuli was also studied.

## 2. Results

### 2.1. Reduction of Infarct Volume in Ischemic Brain

**Figure 1:**
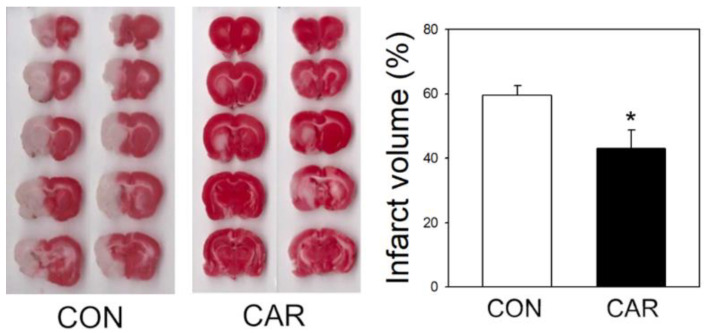
Protective effect of carnosine on infarct volume after focal cerebral ischemic injury in rats. After 3 h of MCAO, rats were administered saline (CON) and carnosine (CAR) intravenously. Cerebral infarct size was identified by TTC staining after ischemic injury in a permanent ischemic model during 24 h and was analyzed by the Image J software program. The ischemic stroke-induced cerebral infarct volume in CON groups (*n* = 15) was reduced in the group treated with CAR (*n* = 15). Data are presented as the mean ± SEM. * *p* < 0.05 vs. CON.

We used middle cerebral artery occlusion (MCAO) to induce ischemic stroke in a rat model to confirm the effect of carnosine on cerebral infarction (Figure 1). During induction of ischemic stroke, cerebral blood flow, temperature, and body weight were monitored and maintained in a recommended range. Rats received carnosine (1000 mg/kg) or saline (control group) intravenously 3 h after MCAO by tail vein. Infarct volume was measured using triphenyltetrazolium chloride (TTC) staining at 24 h. Carnosine treatment at 3 h after ischemic onset significantly reduced cerebral infarction in ischemic stroke, consistent with our previous observations [36,38].

### 2.2. Reduction of Edema

**Figure 2:**
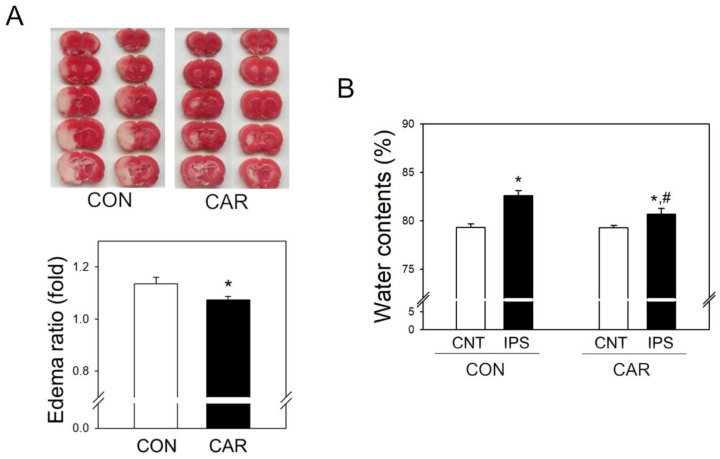
Reduction of cerebral edema and water content in the ischemic brain by carnosine. (**A**) After 24 h of ischemic stroke, the edema ratio of the isolated brain was compared between control and carnosine-treated rats (*n* = 10). Edema ratio was calculated as ischemic hemisphere (ipsilateral)/non-ischemic hemisphere (contralateral). The ratio of edema in the group treated with carnosine (CAR) was decreased, compared to the control group (CON). (**B**) The water content of each hemisphere was also measured 24 h after induction of ischemic stroke (*n* = 5). Each hemisphere was initially weighed immediately after isolation (wet weight). After drying at 95 °C for 24 h, the hemispheres were re-weighed (dry weight). Water content was calculated as ((wet weight − dry weight)/wet weight) × 100 (%). The water content of the ipsilateral area (IPS) in the control group was significantly increased, compared to the contralateral area (CNT). Data are presented as the mean ± SEM. * *p* < 0.05 vs. CON (**A**) and CNT of CON (**B**), # *p* < 0.05 vs. IPS of CON.

We observed that carnosine was protective against brain edema (Figure 2A). In ischemic strokes, brain volume is larger where edema occurs compared to normal brain, as the water content increases and the cells swell [53]. After MCAO, we observed that the ipsilateral hemisphere was more swollen than the contralateral one using TTC staining. Edema ratio was calculated as the ratio of ipsilateral and contralateral hemisphere volume. Edema was significantly reduced in carnosine-treated rats, compared with controls.

### 2.3. Effect of Carnosine on Brain Water Content

Formation of edema with increased brain water content was observed in the early stages of cerebral ischemia in animal models of ischemic stroke and some stroke patients [12,19]. To measure the water content, the brain was isolated and weighed following MCAO. After drying for 24 h, the weight of the brain was measured again, and the water content was calculated (Figure 2B). In both groups, the ipsilateral area showed higher water content than the contralateral area, reflecting the ischemic injury. The water content of the ipsilateral, compared to the contralateral hemisphere, was significantly decreased in the carnosine-treated rats.

### 2.4. Suppression of MMP Activity by Carnosine in Ischemic Brain

**Figure 3:**
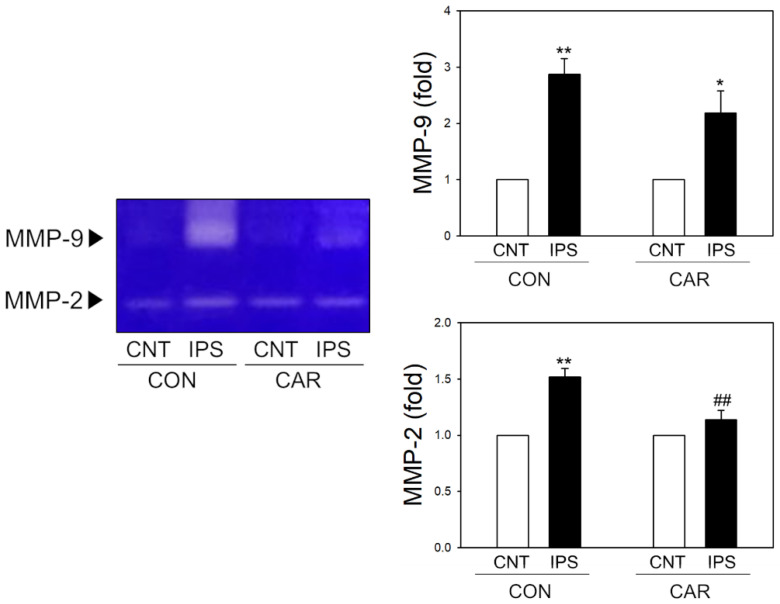
Ex vivo inhibition of MMP activity by carnosine. After the ischemic brain was separated, gelatin zymography was performed to confirm the activity of MMP-2 and MMP-9 (*n* = 5). We measured the activity of MMPs via a non-stained region. The band (non-stained region) was analyzed by the Image J software program. MMP-2 showed significantly increased activity in the ipsilateral area (IPS), compared to the contralateral area (CNT). MMP-2 activity in the ipsilateral area of the carnosine treated group (CAR) was reduced compared to the control group (CON). MMP-9 activity increased in the ipsilateral area in both groups. Data are presented as the mean ± SEM. * *p* < 0.05 vs. CNT of each group, ** *p* < 0.01 vs. CNT of each group, ## *p* < 0.01 vs. IPS of CON.

The activity of MMP-2 and MMP-9 increased in the ischemic brain. It was also associated with edema and destruction of the BBB [40,41]. To measure the MMP activity, we conducted gelatin zymography using brain homogenates following ischemia (Figure 3). Gelatin zymography showed that both MMP-2 and MMP-9 activities were significantly increased in the ipsilateral hemisphere following ischemic stroke. However, post-treatment of carnosine at 3 h after MCAO significantly reduced the activity of MMP-2 in the ipsilateral area compared with the control groups. The activity of MMP-9 was found to increase in the ipsilateral area, but there was no significant difference between the carnosine-treated and the control groups.

### 2.5. Carnosine-Inhibition of MMPs in Enzymatic Assays

**Figure 4:**
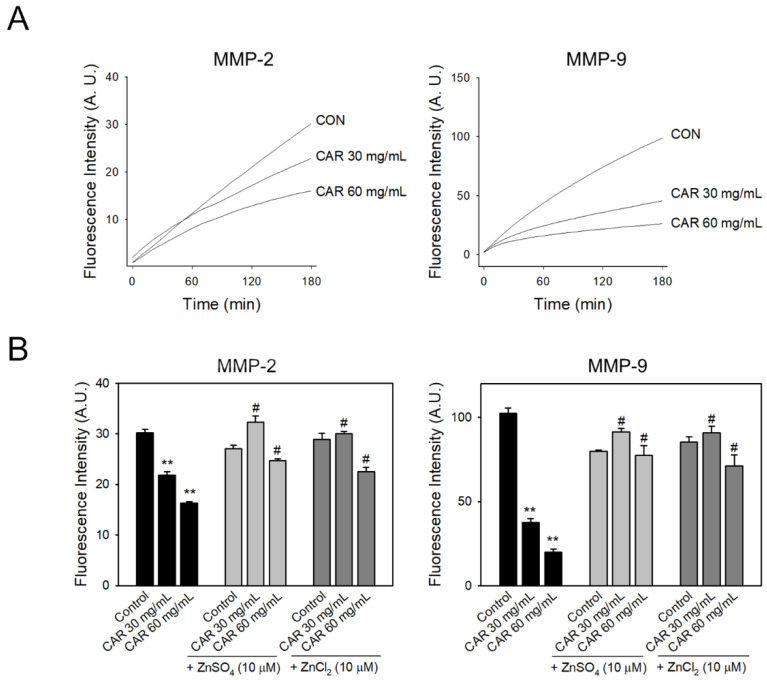
Inhibition of MMP activity by zinc chelation of carnosine. The gelatinase/collagenase assay was used to confirm inhibition of MMP activity by carnosine. (**A**) The activity of MMP-2 and MMP-9 was measured by fluorescence (*n* = 3). In the group treated with carnosine (CAR), the activity decreased compared to the control group (CON) depending on the concentration. Control samples without enzyme were used to determine fluorescence. (**B**) Excessive zinc was added to confirm that carnosine inhibits activity because carnosine is a chelator of zinc (*n* = 5). It was observed that the activity of MMPs significantly reduced by carnosine was increased by the addition of zinc, so carnosine was found to inhibit the activity of MMPs during chelation of zinc. Data are presented as the mean ± SEM. ** *p* < 0.01 vs. control group. # *p* < 0.05 vs. corresponding carnosine-treated group in the absence of excessive amount of zinc.

We observed that carnosine significantly inhibited the activity of MMPs in rat brain homogenates isolated following ischemic stroke. Next, we investigated the direct effect of carnosine on MMP activity using a fluorescent enzymatic assay with recombinant mouse MMP-9 and MMP-2. In both MMP-2 and MMP-9, the carnosine-treated group showed lower fluorescence intensity than the control groups, reflecting that carnosine significantly inhibited the enzyme activity in a dose-dependent manner (Figure 4A).

### 2.6. Inhibitory Effect of Carnosine on Zinc-Mediated MMP Activity 

MMPs are calcium-dependent endopeptidases, containing zinc atoms in the catalytic site [54]. Interestingly, carnosine is known to chelate zinc and copper ions [55]. To investigate if carnosine inhibition of MMP activity was mediated by zinc chelation, we added an excessive amount of zinc and measured the enzyme activity (Figure 4B). The reduction of activities of MMP-2 and MMP-9 in carnosine-treated groups (CAR 30 mg/mL and 60 mg/mL) was significantly restored by the addition of excessive zinc, regardless of the source (ZnSO_4_ and ZnCl_2_), suggesting that carnosine may inhibit MMP activity by chelating zinc required for the enzymatic reaction.

### 2.7. Protective Effect of TJ Proteins by Carnosine in OGD Exposure

**Figure 5:**
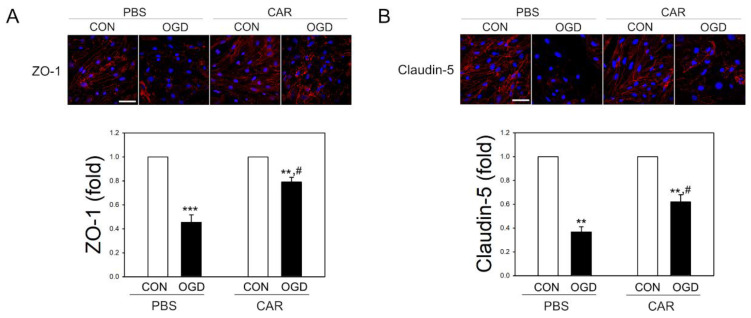
Effect of carnosine on the injury of TJ proteins by OGD exposure in brain endothelial cells. bEnd.3 cells were pretreated with carnosine (CAR) for 18 h before OGD exposure of 6 h. Control cells (CON) were treated with PBS instead of carnosine. The degree of damage to TJ protein after OGD was measured using the ImageJ software program. OGD-exposed bEnd.3 cells were immunostained with an antibody (red) for ZO-1 (**A**) and claudin-5 (**B**) and visualized by confocal microscopy (*n* = 3). Cell nuclei were stained with DAPI (blue). Scale bar: 50 μm. Representative images are shown. Data are presented as mean ± SEM. ** *p* < 0.01 and *** *p* < 0.001 vs. CON of PBS. # *p* < 0.05 vs. OGD of PBS.

We observed decreased activity of MMPs in carnosine treated rats after MCAO. In ischemic stroke, TJ proteins of endothelial cells were disrupted by increased activity of MMPs. In vitro ischemic stimuli of 6 h-OGD significantly reduced the level of TJ proteins, such as ZO-1 and claudin-5 in brain endothelial cells (bEnd.3 cells), as found in confocal microscopy (Figure 5). Of note, when the cells were treated with carnosine (5 mM) for 18 h and then exposed to OGD for 6 h in the presence of carnosine, the extents of ischemic degradation of TJ proteins were significantly reduced.

## 3. Discussion

In the current study, we demonstrated the beneficial effect of carnosine on edema and MMP activation in ischemic stroke. Intravenous administration of carnosine 3 h after MCAO in rats significantly reduced cerebral infarct volume, edema, and brain water content. Carnosine also suppressed MMP activity, which was restored after the addition of excess zinc. Carnosine protects against ischemia induced TJ protein degradation in brain endothelial cells (bEnd.3 cells), indicating that carnosine may protect the BBB from ischemia-induced damage.

Although brain edema does not occur in all stroke patients, it may negatively impact stroke recovery [11,56]. Edema may be initiated in the acute phase of an ischemic stroke and can last for several days, influencing the degree of neurological deficit and extent of recovery [57]. Increased activity of MMPs in ischemic stroke contributes to brain edema and BBB damage [38]. In addition to previous evidence for multimodal protection of carnosine against ischemic stroke, it has provided novel protection potential against brain edema and MMP activation in ischemic conditions [29,31,50,51,52].

In the enzyme assay using recombinant MMPs, carnosine significantly inhibited both MMP-2 and MMP-9 activity (Figure 4). While carnosine significantly reduced MMP-2 activity post-ischemia in rat brain homogenates, there was no statistically significant reduction in MMP-9 activity (Figure 3). This might be due to the relative contribution of different MMPs during ischemic damage while we determined MMP activity at 24 h after ischemic onset with 3 h-post-treatment of carnosine in our rat models. The actions and phases of MMP-2 and MMP-9 differ in the case of an ischemic stroke. MMP-9 is linked to ischemic stroke-induced inflammation and plays a significant role in BBB destruction. MMP-9 activity is high in the acute phase of an ischemic stroke, which increases the risk of bleeding. MMP-2, on the other hand, is important in the later stages of ischemia, during the formation of glial scar within the damaged area [58,59,60,61]. MMP-2 is a constitutive enzyme, whereas MMP-9 is an inducible enzyme. Inducible enzymes are dormant until the neuroinflammation process starts. MMP-2 and MMP-9 play multiple roles due to the complex nature of their interactions with tissues during development, injury, and repair. They participate in the injury process early on and contribute to recovery at later stages. Treatment strategy planning is complicated due to this dual role [62,63]. Our findings are limited because we did not include the various phases of ischemic stroke; however, we observed that carnosine treatment at 3 h after ischemic onset significantly reduced MMP-2 activity, inhibiting edema and cerebral infarction determined at 24 h after ischemia. Further study is warranted to determine the dynamics of inhibitory effects of carnosine on different stages of MMP activation during ischemic stroke.

Carnosine significantly inhibited MMP activity in vitro, which was consistent with the inhibitory effect of carnosine on MMP activity in vivo. As carnosine is known to be a metal chelator, this inhibitory mechanism might be mediated by the chelation of catalytic zinc, which is required for MMP activity [64,65]. The reduced enzymatic activities of MMPs caused by carnosine were found to be restored by the addition of an excessive amount of zinc. These findings suggest that carnosine chelates zinc and thus inhibits MMP activity. Simultaneously, it was demonstrated that excessive zinc treatment had no effect on MMP basal activity (Figure 4B). Given that zinc dysregulation was linked to cell damage and neurotoxicity in a variety of pathological conditions [55], the regulatory effect of carnosine on zinc levels may be beneficial in these circumstances.

Increased MMP activity in endothelial cells damages TJ proteins like ZO-1 and claudin-5, causing BBB damage and brain edema [30,66]. TJ proteins are essential for proper BBB function. However, when the TJ protein is damaged by factors such as ischemic stress and xenobiotics, permeability increases and the brain may not be adequately protected [67,68,69,70]. We used sub-lethal ischemic stimuli of 6 h of OGD that did not significantly reduce cell viability [26], and then observed the degradation of ZO-1 and claudin-5 with a confocal microscope. Carnosine significantly inhibited ischemic TJ degradation while maintaining cell viability, implying that carnosine may protect BBB function.

In conclusion, our findings indicate that carnosine reduces brain edema and MMP activation and adds to the mounting experimental data that supports a robust protective role of carnosine in brain ischemia.

## 4. Materials and Methods

### 4.1. Materials

Carnosine (L-carnosine), gelatin, and TTC (triphenyltetrazolium chloride) were purchased from Sigma-Aldrich (St. Louis, MO, USA). Suture was purchased from Doccol Corp (Redlands, CA, USA). Gelatin-sepharose bead (Gelatin Sepharose 4B) was purchased from GE Healthcare (Chicago, IL, USA). Zymography renaturation buffer, zymography development buffer, and Coomassie Brilliant Blue R-250 was purchased from Bio-Rad (Hercules, CA, USA). Recombinant human MMP-2 was purchased from Merck Millipore (Burlington, MA, USA). Recombinant mouse MMP-9 was purchased from R&D Systems (Minneapolis, MC, USA). EnzChek Gelatinase/Collagenase Assay was purchased from Invitrogen (Carlsbad, CA, USA). Primary antibodies against ZO-1 and claudin-5, Alexa Fluor 555 donkey anti-rabbit, and Pierce BCA protein assay kit were purchased from Thermo Fisher Scientific (Rockford, IL, USA).

### 4.2. Animal Treatment

Adult male Sprague-Dawley rats weighing 235 to 275 g (Harlan: Koatech, Pyeongtaek, Korea) were used in the experiments, and surgical procedures were carried out with the approval of Hanyang University’s Institutional Animal Care and Use Committee (IACUC 2018-0158A, approval date: 9 May 2018). Treatment groups were assigned in a randomized order. The investigators were blind to treatment during surgeries and outcome evaluations. Carnosine was dissolved in saline and administered intravenously (1000 mg/kg) to the tail 3 h after ischemia induction. In the control group, saline was given intravenously to the tail instead of carnosine. The animals were sacrificed, and samples were collected after 24 h of ischemia induction.

### 4.3. Permanent Middle Cerebral Artery Occlusion (pMCAO) in Rat

Intraluminal middle cerebral artery occlusion was used to induce permanent focal ischemia in rats (MCAO). Isoflurane inhalation was used to induce anesthesia, which was maintained throughout the surgery. Rectal temperature was monitored and maintained, both before and during surgery. Before and after the surgery, cerebrovascular blood flow (CBF) was measured using a laser Doppler (Perimed, North Royalton, OH, USA). The left common carotid artery (CCA) and the external carotid artery (ECA) were isolated and sutured tightly. The internal carotid artery (ICA) was separated and the pterygium was coagulated after the ECA branches were cauterized. A silicone-coated 4-0 monofilament nylon suture was inserted into the CCA to induce the ischemia. The suture was advanced, approximately 18 mm through the ICA from the CCA bifurcation to the origin of the MCA.

### 4.4. Triphenyltetrazolium Chloride (TTC) Staining

At 24 h post-MCAO, rats were anesthetized by isoflurane, decapitated, and the brains were carefully and immediately isolated. Brains were cut into 2 mm sections, stained with 2% TTC, and fixed in 4% paraformaldehyde. Each section was used to create a digital image, which was then analyzed using the software program, ImageJ. The hemisphere area for each section was calculated by averaging the measured areas on each side of the section. The ipsilateral/contralateral hemisphere area was used to calculate the edema ratio.

### 4.5. Determination of Water Retention

The percentage of water content in each hemisphere 24 h after induction of ischemic stroke was used to assess edema formation. Hemispheres were initially weighed and recorded as wet weights immediately after isolation. The hemispheres were weighed again and recorded as dry weight after 24 h of desiccation at 95 °C. Water content was calculated as ((wet weight − dry weight)/wet weight) × 100.

### 4.6. Gelatin Zymography

For zymography, hemispheres were homogenized with lysis buffer (50 mmol/L Tris-HCl pH 7.6, 150 mmol/L NaCl, 5 mmol/L CaCl_2_, 0.05% Brij-35, 0.02% NaN_3_, and 1% Triton X-100), including protease inhibitors. The homogenates were centrifuged and the supernatants were collected. The protein concentration in the homogenate was determined using BCA protein assay. Matrix metalloproteinase-2 and -9 in homogenates were concentrated with gelatin-sepharose beads. Equal amounts of samples were electrophoretically separated on 7.5% sodium dodecyl sulfate-polyacrylamide gels with gelatin under nonreducing conditions. The gels were incubated with zymography renaturation buffer twice for 15 min at room temperature. After washing the gel, it was incubated for 30 min at room temperature with zymography development buffer. Then, the gel was incubated with zymography development buffer for 48 h at 37 °C. Following development, the gel was stained for 2 h with Coomassie Brilliant Blue R-250 and then appropriately destained. Proteolytic bands in zymography were measured by ImageJ software program.

### 4.7. In Vitro MMP Activity Assay

Recombinant human MMP-2 and recombinant mouse MMP-9 were activated with p-aminophenylmercuric acetate (APMA), as recommended by the manufacturer. Enzyme activity was measured using the EnzChek Gelatinase/Collagenase Assay following the manufacturer’s protocol, with detection on a fluorescent plate reader. Control samples without enzyme were used to determine background fluorescence.

### 4.8. Cell Culture

The bEnd.3 cell line, immortalized mouse brain endothelial cells, was obtained from American Type Culture Collection (Manassas, VA, USA). bEnd.3 cells were grown in DMEM (Dulbecco’s Modified Eagle’s Medium with 4500 mg/L d-glucose, 110 mg/L sodium pyruvate, 1.5 g/L sodium bicarbonate, and l-glutamine; Welgene, Daegu, Korea), supplemented with 10% fetal bovine serum (FBS; Mediatech Inc., Manassas, VA, USA), 100 units/mL of penicillin, and 100 μg/mL of streptomycin (Welgene, Gyeongsan, Korea). bEnd.3 cells were maintained in the incubator at 37 °C, with 5% CO_2_ and 95% air. All experiments were carried out when the density was 90–100%.

### 4.9. Oxygen-Glucose Deprivation (OGD)

Before OGD, cells in the control and carnosine groups were pretreated for 18 h with PBS or 5 mM carnosine, respectively. The OGD group was added to DMEM without D-glucose and FBS, and the control group was washed twice with DMEM without FBS. In addition, for OGD stimulation or control treatment, the media were replaced with glucose-free DMEM or DMEM supplemented with 5.5 mM glucose and did not include FBS. Cell plates were placed in a hypoxia chamber (Billups-Rothenberg Inc., San Diego, CA, USA), and the air was replaced with OGD gas (95% N_2_ and 5% CO_2_). Cells were subjected to the OGD condition for 6 h at 37 °C to measure the TJ proteins. Oxygen depletion in the chamber was monitored using BD GasPak™ Dry Anaerobic Indicator Strips (BD, Franklin Lakes, NJ, USA).

### 4.10. Immunofluorescence Staining

Cells were seeded into a Lab-Tek™ 8-well Chambered Coverglass (Thermo Fisher Scientific). OGD was performed on the cells once they had reached confluency. Cells were fixed and permeabilized with ice-cold methanol and acetone for 10 min and blocked using 5% normal donkey serum (Sigma-Aldrich) in PBS for 1 h. The cells were incubated with primary antibodies (anti-ZO-1 and anti-claudin-5 antibodies) diluted in 1% normal donkey serum and then further incubated with secondary antibodies (Alexa Fluor 555-conjugated anti-rabbit) in 1% BSA. Fluorescence images were acquired and analyzed with a K1-Fluo confocal laser scanning microscope (Nanoscope Systems, Daejeon, Korea). To quantify the degree of TJs proteins, fluorescence images were analyzed with the software program, ImageJ.

### 4.11. Statistics

All experimental values were expressed as the mean and standard error (SEM). Statistical significance between groups was determined by the Student’s t-test. In all analyses, a *p* value < 0.05 was considered statistically significant.

## Data Availability

Data is contained within the article.
